# Properdin Modulates Complement Component Production in Stressed Human Primary Retinal Pigment Epithelium Cells

**DOI:** 10.3390/antiox9090793

**Published:** 2020-08-26

**Authors:** Nicole Schäfer, Hannah N. Wolf, Anne Enzbrenner, Juliane Schikora, Maria Reichenthaler, Volker Enzmann, Diana Pauly

**Affiliations:** 1 Experimental Ophthalmology, Eye clinic, University Hospital Regensburg, 93053 Regensburg, Germany; Nicole.Schaefer@vkl.uni-regensburg.de (N.S.); Hannah.Wolf@stud.uni-regensburg.de (H.N.W.); A.enzbrenner@googlemail.com (A.E.); Juliane.Schikora@stud.uni-regensburg.de (J.S.); Maria.reichenthaler@googlemail.com (M.R.); 2 Department of Ophthalmology, University Hospital of Bern, University of Bern, 3010 Bern, Switzerland; Volker.Enzmann@insel.ch; 3 Department of Biomedical Research, University of Bern, 3010 Bern, Switzerland

**Keywords:** retinal pigment epithelium, complement system, properdin, AMD-risk genotype, intracellular, cell-associated, inflammasome, oxidative stress, human

## Abstract

The retinal pigment epithelium (RPE) maintains visual function and preserves structural integrity of the retina. Chronic dysfunction of the RPE is associated with retinal degeneration, including age-related macular degeneration (AMD). The AMD pathogenesis includes both increased oxidative stress and complement dysregulation. Physiological sources of oxidative stress in the retina are well known, while complement sources and regulation are still under debate. Using human primary RPE (hpRPE) cells, we have established a model to investigate complement component expression on transcript and protein level in AMD-risk and non-risk hpRPE cells. We evaluated the effect of properdin, a complement stabilizer, on the hpRPE cell-dependent complement profile exposed to oxidative stress. hpRPE cells expressed complement components, receptors and regulators. Complement proteins were also stored and secreted by hpRPE cells. We associated AMD-risk single nucleotide polymorphisms with an increased secretion of complement factors D (CFD) and I (CFI). Furthermore, we detected hpRPE cell-associated complement activation products (C3a, C5a) independent of any extracellularly added complement system. Exogenous properdin increased the mRNA expression of *CFI* and *CFD*, but decreased levels of complement components (*C1Q*, *C3*), receptors (*C3AR*, *C5AR1*, *CD11B*) and inflammation-associated transcripts (*NLRP3*, *IL1B*) in hpRPE cells exposed to oxidative stress. This properdin effect was time-dependently counter regulated. In conclusion, our data unveiled a local, genotype-associated complement component production in hpRPE cells, regulated by exogenous properdin. The local complement production and activation via blood-independent mechanisms can be a new therapeutic target for AMD.

## 1. Introduction

As early as 2005, single nucleotide polymorphisms (SNP) in the *complement factor H (CFH)* gene were identified as genetic risk factors for age-related macular degeneration (AMD), the major cause of visual impairment in the Western world [1,2]. Today, it is known that at least eight of these AMD-risk factors reside in different genes encoding the complement system and enhanced complement deposition was observed in AMD-affected eyes [3,4,5,6]. However, we still miss a satisfactory answer how these SNPs or the complement system as a whole contributes to AMD.

The complement system is a pathway of the innate immune system, consisting of over 40 proteins, which are consecutively activated. Properdin, is the only known stabilizer of the complement system [7]. It binds to the central, activating protein complex of the cascade and prolongs its half-life by 5–10 times. Next to stabilizing the central C3 convertase, properdin has also a potential role as a pattern recognition molecule activating the complement pathway. The whole complement cascade ensures a first line defense against pathogens and modified cells producing alarm molecules (anaphylatoxins), tagging cells/microorganisms (opsonins) or disrupting cell membranes (membrane attack complex) [8]. Additionally, non-canonical intracellular functions of complement components (“the complosome”) have been described in T-cells, neutrophils, pancreatic β-cells and others [9,10,11]. Cell-associated or intracellular complement activity modulated cell metabolism, autophagy, survival, and differentiation in these different cell types [10,12,13,14]. However, so far the complement system has not been further investigated as a cell-dependent/autocrine pathway in relation to AMD so far.

Two major advanced stages of AMD can occur simultaneously in one patient or even in a single eye: Choroidal neovascularization (CNV) and geographic atrophy (GA) [15,16]. These completely different disease patterns cause either disruption or loss of the retinal pigment epithelium (RPE). Besides genetics, clinical data suggested additional external stimuli, for example oxidative stress or aging processes [17,18], promoting different pathological outcomes in AMD. This needs to be taken into account investigating the role of complement in RPE and AMD.

The RPE forms the blood–retinal barrier, which separates the retina from the systemic circulation and the immune system [19]. The RPE acts as a regulatory, secretory epithelium supporting the retina. It locally secretes complement components as C1q, complement factor B (CFB), complement component 4 (C4), CFI, and CFH [20,21,22,23]. We and others showed that complement secretion is modified by external stress [20,21,22,23,24,25,26]. Additionally, generation of complement activation products, such as anaphylatoxins and opsonins, by healthy and stressed RPE cells independent of any external complement source is described [21,24,26,27]. Recently, it was reported, that endogenous CFH and anaphylatoxins contribute to transcriptional and metabolic homeostasis of RPE cells [28,29,30]. In RPE cells complement anaphylatoxins receptor signaling is involved in eye morphogenesis [31], sub-RPE deposits [32], pro-inflammatory RPE reaction [33,34,35], PI3/Akt-pathway activation [29], and stress-mediated lipid accumulation in RPE cells [36]. Together this indicates an involvement of autocrine complement reactivity in housekeeping mechanisms maintaining RPE physiology. However, it is not known in detail how this is controlled and how it contributes to retinal degeneration.

In the present study, we tested whether human primary RPE (hpRPE) cells produce and activate complement components in dependence of their genotype and exogenous properdin stress. We demonstrated that hpRPE cells positive for a homozygous AMD-risk SNP within complement genes secreted more complement proteins than non-carriers. Thereby, we supposed that the complement stabilizer properdin modifies the local complement homeostasis in stressed hpRPE cells. We described that hpRPE cell-dependent complement levels were time-dependently changed by oxidative stress and properdin addition.

## 2. Materials and Methods

### 2.1. Cultivation and Treatment of hpRPE

The research complies with the human research act (HRA) stating that small quantities of bodily substances removed in the course of transplantation may be anonymized for research purposes without consent (HRA chapter 5, paragraph 38, Switzerland). hpRPE were prepared from left and right eyes of 15 anonymized donors (Table 1) as previously described [37,38]. Briefly, hpRPE cells were harvested from the eyecup after enzymatic digestion and centrifuged with 259× g at 4 °C for 5 min. hpRPE cells were cultivated in Dulbecco’s modified Eagle’s medium/Nutrient Mixture F-12 (DMEM/F 12 GlutaMax, Thermo Fisher Scientific, Dreieich, Germany, #31331-028) containing 5% fetal bovine serum (FBS, Thermo Fisher Scientific, #10500-064), 1% Penicillin-Streptomycin (Thermo Fisher Scientific, #15070-063), 1% N1 Medium Supplement (Merck, Darmstadt, Germany, #N6530), 10 mM MEM non-essential amino acids (Thermo Fisher Scientific, #11140-035), 0.25 mg/mL Taurine (Merck, #T0625), 4.5 mg/mL Glucose solution (Thermo Fisher Scientific, #G524940-01), 0.013 ng/mL Triiodothyronine (Merck, #T2877), 0.02 µg/mL Hydrocortisone (Merck, #H0888), 20 ng/mL human basic growth factor (hbFGF, R&D Systems, Minneapolis, MN, USA, #13256029) and 1 mg/mL human epidermal growth factor (hEGF, Thermo Fisher Scientific, #PHG0311) in laminin-coated Transwell^®^ inserts under standard conditions (37 °C, 5% CO_2_, 80% humidity).

Before treatment, FBS concentration was reduced to 0% within 3 days (5%–2.5%–1.25%, respectively). hpRPE cells were apically treated either with 0.5 mM H_2_O_2_, or 0.5 mM H_2_O_2_ and 50 µg/mL properdin (Complement Technology, Tyler, TC, USA, #A139) for 4, 10, or 24 h. Supernatants (apical and basal) were taken before and after treatment, subsequently frozen in liquid nitrogen and stored at −80 °C.

### 2.2. hpRPE Genotyping

DNA was isolated from sclera slices of the respective donor eyes using ReliaPrep™ FFPE gDNA Miniprep System (Promega, Mannheim, Germany, #A2351). PCR for amplification of DNA sequences containing relevant AMD-associated complement SNPs was performed using in-house generated primers (Table S1) and the following cycle steps, according to the MIQE guidelines [39]: denaturation (95 °C, 1 min), annealing (60 °C, 1 min), elongation (72 °C, 1 min), 33 cycles. Afterwards, DNA-sequencing was performed by GeneArt (Thermo Fisher Scientific) using either forward or reverse in-house primers, respectively (Table S1).

### 2.3. RT-qPCR and PCR

mRNA was isolated using the NucleoSpin^®^ RNA kit (Macherey-Nagel, Düren, Germany, #740955) and transcribed into cDNA with the QuantiTect^®^ Reverse Transcription Kit (Qiagen, Hilden, Germany, #205313). Transcripts of complement components, regulators, receptors and inflammation-associated markers were analyzed (i) by PCR (as described in Section 2.2.) and subsequent 2% agarosegel separation for cDNA fragment visualization, or (ii) by qPCR using Rotor-Gene SYBR^®^ Green PCR Kit (Qiagen) in a Rotor Gene Q 2plex cycler (Qiagen) either with QuantiTect Primer Assays (Qiagen, Table S2), or in-house designed primers for *IL1B*, *VIM* and *SMA1* (Metabion, Planedd, Germany, Table S1). Data were normalized to the housekeeper GAPDH expression and fold change was calculates using 2(-Delta Delta C(T)) method [40]. Data are visualized on a linear scale using log2-transformed scores [41].

### 2.4. Multiplex Complement Secretion Assay

Complement components in the cellular supernatants after 11–14 days of cultivation were quantified using the MILLIPLEX MAP Human Complement Panel (Merck, #HCMP1MAG-19K, #HCMP2MAG-19K) in accordance to the manufacturer’s protocol. The read out of the multiplex assay was performed in a Magpix instrument (Luminex, Austin, TX, USA).

### 2.5. Western Blot

Lysates of hpRPE cells were generated using RIPA buffer (Sigma-Aldrich, Munich, Germany, #R0278) with protease and phosphatase inhibitors (1:100, Sigma-Aldrich, #P8340). Samples were dissolved in reducing Laemmli sample buffer, denatured (95 °C, 10 min) and separated in a 12% SDS-PAGE and transferred on to an activated PVDF membrane using a wet blotting system. Membranes were blocked (1 h, 5% bovine serum albumin (BSA)/PBS-T) and incubated with the primary antibodies (overnight, 5% BSA/PBS-T): Rabbit anti-GAPDH-HRP (1:1000, Cell Signaling Technology, Beverly, MA, USA, #3683), mouse anti-C3 (1:100, Progen, Heidelberg, Germany, #61019), goat anti-CFI (1:250, Quidel, San Diego, CA, USA, #A313), mouse anti-C3a (1:50, Hycult, Uden, Netherlands, #HM2074) and mouse anti-C5a-biotin (1:250, Biozol, Eching, Germany, #BLD-518306). Peroxdiase-conjugated anti-species antibodies were used for detection (1 h, PBS-T): goat anti-mouse (1:5000, Jackson ImmunoResearch, West Grove, PA, USA, #115-035-164), rabbit anti-goat (1:5000, Jackson ImmunoResearch, #305-035-003). Visualization was performed by WesternSure PREMIUM Chemiluminescent Substrate (LI-COR, Bad Homburg, Germany, #926-95000) in a Fluor Chem FC2 Imaging System (Alpha Innotech, San Leandro, CA, USA).

### 2.6. Immunohistochemistry

Immunostaining was performed as described previously [22]. PBS-washed, paraformaldehyde-fixated (4%, 20 min; Merck, # 100496) hpRPE cells were blocked 3% BSA (Carl Roth, Karlsruhe, Germany, #8076/ PBS-T, 1 h). Antigens were detected using primary antibodies anti-RPE65 (1:200; Abcam, Cambridge, UK #ab13826) or anti-bestrophin (1:200; Novus Biologicals, Littleton, CO, USA #NB300-164), visualized with goat anti-mouse ALEXA Fluor 594 (1:500; Thermo Fisher Scientific, #A32742) and complemented with nuclei staining (DAPI; Vector Labs, Burlingame, CA, USA, #H-2000,). Cells were covered with fluorescence mounting medium (Agilent, Boeblingen, Germany, #S302380-2).

### 2.7. Statistics

Statistical analysis was performed using GraphPad Prism 7 (GraphPad Software Inc, San Diego, CA, USA). We estimated a normal Gaussian distribution. Significance levels were determined by the parametric, unpaired *t*-test. Welch‘s test was used to correct for unequal sample distribution variance. All data are expressed as mean ± standard deviation (SD) unless stated otherwise. Detailed information about specific n-values, implemented statistical tests and coding of significance levels are provided in the respective figures and figure legends.

## 3. Results

RPE cells are capable of producing cell-derived complement components as we showed recently [22,42]. In the present study we assume that an externally added complement regulator, properdin, can modulate stress-dependent RPE cell-derived complement components.

### 3.1. Human Primary RPE Cells as a Model System in Cell Culture

We investigated human primary RPE (hpRPE) cells, isolated and cultivated from 15 different donor eyes (Table 1). Studied hpRPE cells were vital and pigmented as well as positive for the RPE markers RPE65 and bestrophin (Figure S1A–G). Pigmentation of the hpRPE cells was stable from 4 to 14 days of cultivation (after removal of non-adherent cells at day 2).

Ex vivo cultivation of hpRPE of both eyes resulted in a maximum of 6 transwell filters with a growth area of 1.12 cm^2^ from each donor. This limited the study and not all investigations could be performed with the hpRPE cells from the same donor. An overview of the used cell preparations and experiments is given in Table 1.

### 3.2. hpRPE Cells Produce Complement Components

**Figure 1:**
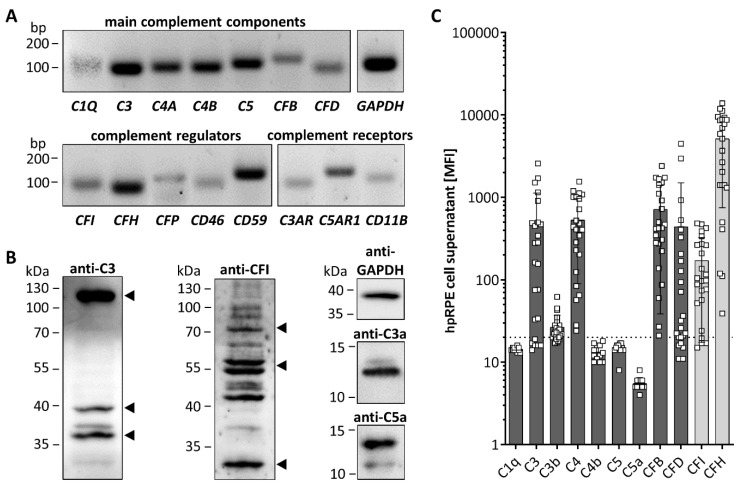
hpRPE cells express and secrete complement components. (**A**) mRNA expression of main complement components of the classical (*C1Q*), central (*C3*) classical/lectin (*C4A*, *C4B*), terminal (*C5*) and alternative (*CFB*, *CFD*) complement pathway was detected in hpRPE cells. hpRPE cells expressed also mRNA of soluble (*CFI*, *CFH*, *CFP*) and membrane bound (*CD46*, *CD59*) complement regulators, and transcripts of complement receptors: anaphylatoxin receptors (*C3AR*, *C5AR1*), opsonin receptor CR3 subunit (*CD11B*). Shown expression data of donor 1 are representative for mRNA experiments in hpRPE cells of donors 1–4 (Table 1). (**B**) Activation products of the central complement component C3 [(blot left, arrows) C3b (115 kDa), C3dg (39 kDa) and C3d (35 kDa)] were detected in hpRPE. Multiple bands were identified in hpRPE cells using an anti-complement regulator CFI antibody (blot center, arrows for full CFI (80 kDa) and two CFI disulfide linked chains (50 kDa, 30 kDa)). Anaphylatoxins C3a and C5a (blot right) were found in cell lysates of hpRPE cells. Examples of whole blots are depicted in **(B)** for donor 5 (C5a) and donor 6 (C3, C3a, CFI). (**C**) hpRPE cells secrete C3, C4, CFB, CFD and the regulators CFI as well as CFH into the cell culture supernatant (pooled data of two eyes, apical and basal supernatant are shown, details for hpRPE cells of donors 11–16 are presented in Figure S3). Mean with standard deviation is shown. Dotted line depicts blank control. MFI—mean fluorescence intensity.

All tested hpRPE cells were positive for transcripts of complement components *C1Q*, *C3*, *C4A*, *C4B*, *C5*, *CFB*, and *CFD*, as well as complement regulators *CFI*, *CFH*, *properdin* (*CFP)*, *CD46,* and *CD59* (Figure 1A). hpRPE cells expressed also complement receptors for complement activation products like the anaphylatoxin receptors *C3AR* and *C5AR1* as well as the subunit *CD11B* of complement receptor 3, which interacts with the C3 cleavage products (iC3b, C3d, C3dg) upon activation (Figure 1A).

hpRPE cells produced complement proteins as C3 and CFI shown by Western blot (Figure 1B). This raised the question, if complement receptors of hpRPE cells could be modulated in an autocrine signaling pathway if complement activation products are generated by these cells. Analyzing hpRPE cell lysates in Western blot, we found hpRPE cell-derived complement activation products C3a, C5a and further C3 cleavage products (C3d, C3dg) in untreated hpRPE cells independent from any external complement sources, indicating a possible autocrine cleavage and function of hpRPE cell-derived activated complement components (Figure 1B and Figure S2 ).

We also determined secreted complement components in the cell culture supernatants of hpRPE preparations from both eyes 11–14 days after isolation (Figure 1C). Thereby, we detected C3, C3b, C4, CFB, CFD and the regulators CFI as well as CFH in the supernatants (Figure 1C). C1q, C5 and the complement activation products C4b and C5a were either not secreted or below the assay detection limits (Figure 1C). We also identified differences of complement secretion levels between hpRPE cell preparations of the left and right eye (Figure S3). However, we cannot judge the influence of retinal phenotype and cell preparation differences. Apical and basal complement secretion levels were comparable (Figure S3), but due to low cell numbers and reduced cell division of hpRPE a confluent monolayer was not always observable. Furthermore, we cannot exclude passive diffusion of the complement components secreted apically and basally through the membrane filter (Figure S3).

### 3.3. AMD-Risk SNPs in Complement Genes Increase hpRPE Cell-Dependent Complement Secretion

SNPs within genes of the complement cascade were previously associated with the risk of retinal degeneration and changed retinal/RPE physiology [4,43,44]. We analyzed 13 complement-associated risk SNPs in the donor tissues to evaluate the effect of these SNPs on hpRPE cell-derived complement secretion. We found 4 of 15 donors positive for 1–3 homozygous AMD-risk SNPs either in the *CFH*, *C2/CFB* or *CFI* gene loci and all donors were positive for 1–5 heterozygous AMD-risk SNPs (Table 1).

**Figure 2:**
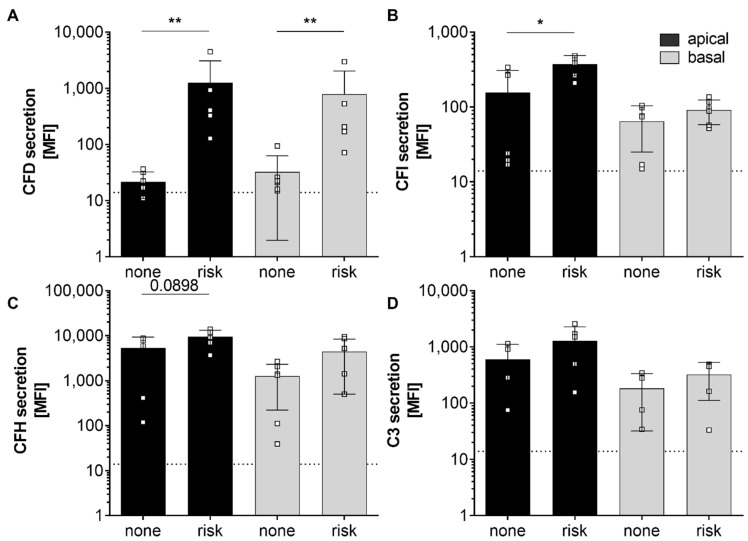
Secretion of alternative complement pathway components was increased in hpRPE cells positive for homozygous age-related macular degeneration (AMD)-risk single nucleotide polymorphisms (SNPs) (risk; donors 12, 13, 15). Significant higher levels of secreted (**A**) complement factors D (CFD), (**B**) complement factors I (CFI) and in tendency higher (**C**) complement factor H (CFH) and (**D**) C3 secretion levels were detected in apical (black) and basal (grey) supernatants of hpRPE cells with homozygous AMD-risk SNPs (risk; donors 12, 13, 15) compared to hpRPE cells without a homozygous AMD-risk SNP (none; donors 10, 11, 14) in complement genes (Table 1). Mean with standard deviation is shown. * *p* < 0.05, ** *p* < 0.01 unpaired *t*-test with Welch‘s correction. Dotted line depicts blank control. MFI—mean fluorescence intensity.

Furthermore, we compared complement secretion levels of hpRPE cells positive for at least one homozygous AMD-risk SNPs SNP (donors 12, 13, 15) and non-homozygous AMD-risk SNP hpRPE cells (donors 10, 11, 14) (Table 1, Figure 2). Thereby, significantly higher CFD and CFI levels in supernatants of hpRPE cells with the AMD-associated risk SNPs in complement genes were found (Figure 2A,B). A comparable tendency was also observed for complement regulators CFH and the central complement component C3, which were increased in supernatants of hpRPE with the AMD-risk genotypes after 14 days of cultivation compared to non-risk cells (Figure 2C,D). Only the secretion of components of the alternative pathway was affected by AMD-risk SNPs in complement genes under the described experimental conditions. However, we cannot definitely state that the hpRPE culture always resulted in the same cell numbers on the transwell filters investigated (even the same preparation protocol was used).

### 3.4. Complement Regulator Properdin Increased Expression of Complement Proteases in Stressed hpRPE Cells

**Figure 3:**
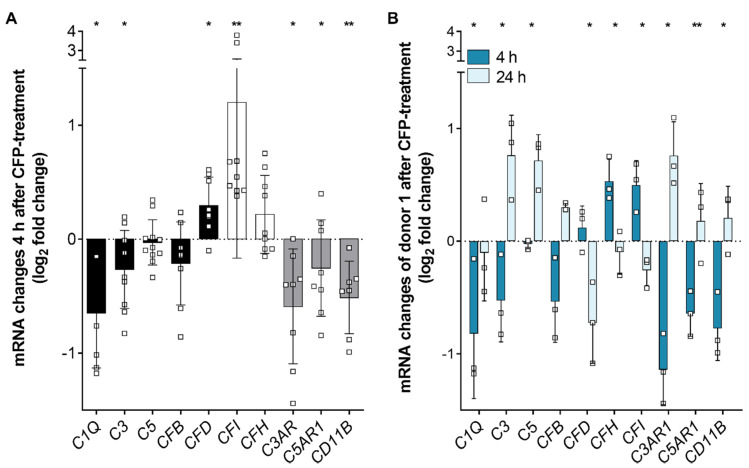
Complement regulator properdin changed complement mRNA expression in stressed hpRPE cells. (**A**) H_2_O_2_ stressed hpRPE cells were treated with properdin (CFP) for 4 h. mRNA expression of main complement components *C1Q*, *C3*, *C5*, and *CFB* (black) as well as complement receptors *C3AR*, *C5AR1*, and *CD11B* (grey) was decreased in comparison to stressed cells (dotted line). *CFD* (black) and complement regulators *CFI* and *CFH* (white) mRNA expression was increased in stressed, properdin-treated cells compared to stressed cells only. Data from donors 1–4 are presented. Mean with standard deviation is shown. * *p* < 0.05, ** *p* < 0.01 unpaired *t*-test with Welch‘s correction of stressed versus stressed, properdin-treated cells. (**B**) H_2_O_2_ stressed hpRPE cells of donor 1 were treated with properdin for 4 h (dark blue) or 24 h (light blue). mRNA Expression of complement components and receptors was reversed after 24 h compared to 4 h. Mean with standard deviation is shown. * *p* < 0.05, ** *p* < 0.01 unpaired *t*-test with Welch‘s correction of 4 h versus 24 h properdin treatment.

We showed recently that oxidative stress increased the expression of complement regulator properdin in RPE cells [22]. In the present study, we investigated the effect of externally added properdin on stressed hpRPE cells. hpRPE cells were treated either with H_2_O_2_ alone or with combined H_2_O_2_ and properdin. Transcript levels of markers for epithelial–mesenchymal transition (EMT), complement components, regulators and receptors were compared between these treatment groups (Figure 3 and Figure S1). After 4 h of properdin treatment, hpRPE cells showed a tendency of increased EMT markers (Figure S1). Simultaneously, transcripts for complement proteases *CFD* and *CFI* were significantly upregulated in hpRPE cells (Figure 3A), while other complement components, which are involved in the general complement cascade (*C1Q*, *C3*), and complement receptors (*C3AR*, *C5AR1*, *CD11B*) were downregulated (Figure 3A). This effect slightly differed in the four tested hpRPE preparations of donors 1–4 pooled in herein, but a genotype-dependent reaction was not observable due to limitations of the cohort size. All tested hpRPE preparations were negative for a homozygous AMD-risk SNP within complement genes (Table 1). Properdin treatment of stressed cells did not change the expression levels of *C4*, *CD59*, *CD46,* and *CFP* (data not shown).

hpRPE cells of donor 1 were used to compare the early (4 h) and late (24 h) effect of external properdin on transcription levels (Figure 3B). We found time-dependent differences of complement expression in properdin-treated cells for all investigated transcripts. Transcription tendencies detected after 4 h were always reversed after 24 h. For example, complement proteases *CFD* and *CFI* were decreased and mRNA of complement receptors *C3AR*, *C5AR1*, and *CD11B* were increased after 24 h (Figure 3B).

In summary, our expression data suggested an early anti-inflammatory and a late pro-inflammatory transcriptional response of stressed hpRPE cells upon properdin treatment.

**Figure 4:**
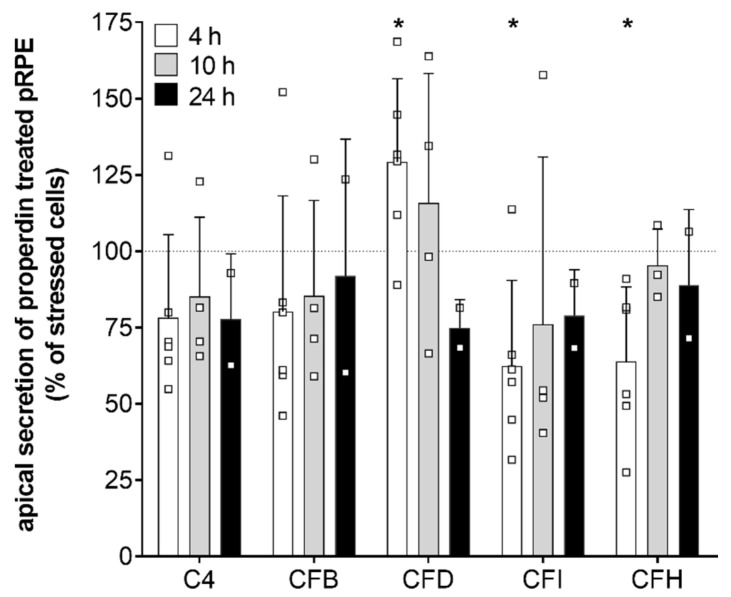
Complement protein secretion was modulated in stressed hpRPE cells by properdin. Complement protein secretion into the apical supernatant 4 h (white), 10 h (grey) and 24 h (black) after properdin treatment of H_2_O_2_ stressed hpRPE is shown as percentage of the secretion level of stressed, non-properdin treated cells (dotted line). We observed a time-dependent change in CFD levels (significantly increased after 4 h) in apical supernatants of stressed properdin-treated hpRPE cells. Decreased concentrations of complement regulators CFI and CFH were detected after 4 h of properdin treatment. Shown are data of donors 2, 4, and 8 for 4 h; donors 3, 5, and 6 for 10 h; donor 1 for 24 h. Mean with standard deviation is shown. * *p* < 0.05, ** *p* < 0.01 *t*-test with Welch‘s correction of stressed hpRPE versus stressed, properdin-treated hpRPE.

Consistent with the transcription results secretion of complement components CFD, CFI, and CFH was time-dependently changed in stressed, properdin-treated hpRPE cells compared to stressed cells (Figure 4). CFD levels in the apical supernatant were increased 4 h after properdin treatment and declined over time. This was in accordance with the mRNA transcription analyses (Figure 3). In contrast to the transcription results, where we described an early increase of CFI and CFH mRNA, the protein levels of CFI and CFH were rather reduced in the supernatant of properdin-treated hpRPE cells (Figure 4). This suggested an intracellular accumulation of these proteins following properdin interaction as described previously for stressed ARPE-19 cells [22]. C4 and CFB secretion was not significantly changed by properdin addition (Figure 4). C3 secretion could not be reliably determined, as the added properdin resulted in unspecific signals in the used commercial multiplex complement assay.

### 3.5. Inflammasome-Associated Transcription Levels were Reduced by Properdin in Stressed hpRPE Cells

**Figure 5:**
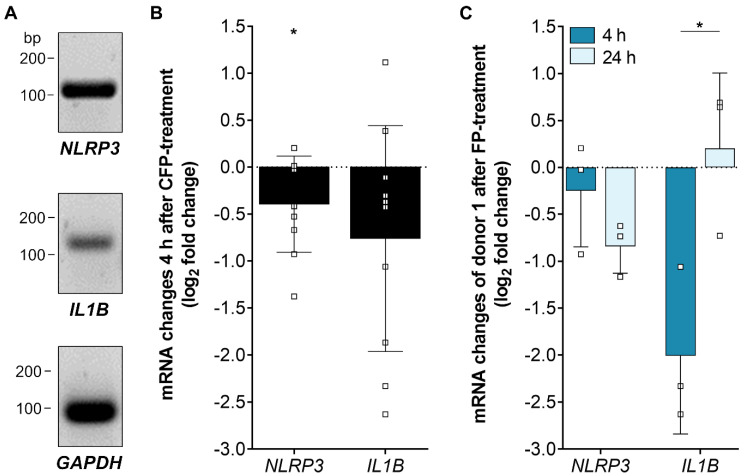
Inflammasome-associated mRNA transcripts were differentially expressed in stressed, properdin-treated hpRPE cells. (**A**) Inflammasome *NLRP3* and pro-inflammatory cytokine *IL1Β* mRNA was detected in hpRPE cells. Shown expression data of donor 1 are representative for mRNA experiments in hpRPE cells of donors 1–4 (Table 1). (**B**) Properdin treatment of H_2_O_2_-stressed hpRPE cells showed a significant reduced expression of *NLRP3* and *IL1Β* after 4 h. Data from donors 1–4 are presented. (**C**) H_2_O_2_-stressed hpRPE cells of donor 1 were treated with properdin for 4 h (dark blue) or 24 h (light blue). Reduced *NLRP3* mRNA expression was accelerated over time and *IL1Β* transcripts were significantly increased after 24 h compared to 4 h properdin incubation. Mean with standard deviation is shown. * *p* < 0.05, ** *p* < 0.01 unpaired *t*-test with Welch‘s correction of 4 h versus 24 h properdin treatment.

Recently, we showed an increase of inflammasome activation following oxidative stress in ARPE-19 cells [22]. To answer the question how the complement regulator properdin acts on inflammasome-associated mRNA expression in stressed RPE cells, we investigated *NLRP3* and *IL1Β* transcripts in properdin-treated and non-treated stressed hpRPE cells (Figure 5). *NLRP3* and *IL1Β* expression was decreased after 4 h in properdin-treated cells compared to only H_2_O_2_-stressed cells (Figure 5B). This confirmed the suggested early anti-inflammatory effect of properdin on H_2_O_2_-stressed hpRPE cells (Figure 3A). After 24 h *NLRP3* transcripts were still reduced, but *IL1Β* were counter regulated in comparison to 4 h with increased *IL1Β* levels (Figure 5C).

## 4. Discussion

AMD is a multifactorial pathological process in the retina resulting from alteration of the RPE. Chronic inflammatory changes in the RPE can lead to blood–retinal barrier breakdown and disease progression. Thereby, the innate immunity including the complement system plays a pivotal role. Our study demonstrated that components of the complement system, a part of the innate immune system are produced and secreted by RPE cells. The local complement quantity and activity was modulated by external stress affecting the RPE and contributing to a disturbed blood–retinal barrier.

### 4.1. Biological Relevance of hpRPE In Vitro Experiments

RPE cells are differentiated and polarized cells, which form a monolayer and are part of the blood–retinal barrier between the photoreceptors and Bruch’s membrane. The main functions of the RPE are: (i) Absorption of light and protection against photo-oxidation, (ii) secretion of immune modulators and growth factors as well as (iii) phagocytosis and recycling of shed photoreceptor [19]. These activities can be altered under non-physiological conditions. In this study, we used hpRPE cells from human donors in order to assure as much in vivo-like RPE cell characteristics as possible in our in vitro experiments. Different genotypes, environmental influences and in vivo differentiation had an impact on the variability and biological relevance of our results. We investigated hpRPE cells from the right and the left eye of 15 different donors, which reflected the RPE phenotype in the human eye [45]. This attached high importance to our results compared to results obtained from immortal, manipulated cell lines [46,47,48] (RPE in vitro systems were excellently reviewed in [19]). Optimal culture conditions resulted in physiological cell morphology and pigmentation of the ex vivo supplied cells [45]. Pigmentation of the RPE cells was a marker for differentiation, not often detected in cultured human RPE cell lines [49].

### 4.2. hpRPE as a Source for Complement Components in the Eye

Previous work identified the RPE as one of the major sources of complement transcripts in the mouse retina [42,50]. Here, we presented for the first time a comprehensive description of transcribed complement components (*C1Q*, *C3*, *C4*, *C5*, *CFB*, *CFD*), regulators (*CFI*, *CFH*, *CFP*, *CD46*, *CD59*) and receptors (*C3AR*, *C5AR1*, *CD11B*) in cultivated hpRPE cells. In accordance with our results *C3*, *CFH*, and complement receptor *C5AR* have already been shown to be expressed in the immortalized retinal pigment epithelial cell line ARPE-19 and in primary RPE cells [22,35,51]. RPE cells derived from induced pluripotent stem cells (iPSC) express mRNA for complement activators *C3*, *C5*, *CFB,* and complement inhibitors *CFH*, *CFI*, *CD46*, *CD55*, *CD59*, *CLUSTERIN*, and *VITRONECTIN* [23]. Single cell transcriptomic analysis in human organoids also showed *CFH*, *C3*, and *C2* expression in RPE cells [52].

Similar to hpRPE, non-liver dependent complement gene expression was also described for intestinal epithelial cells, podocytes and other cell types [42,53,54].

In addition to complement transcripts, we also detected complement components C3, C3b, C4, CFB, CFD as well as complement regulators CFI and CFH in supernatants of hpRPE cells independent of any external complement sources. This was in line with previously published data for post-confluent ARPE-19 cells which secreted C3, C3a, C4bp, CFI, and CFH [20,21,22] and iPSC-derived RPE cells that secreted CFB [23,26]. C3 was previously also found in supernatants of primary mouse RPE cells [50]. Therewith, our and other results suggest a local RPE cell-dependent source of the complement components independent from liver-derived complement proteins in the eye.

hpRPE cell-dependent complement secretion was regulated by environmental, external stimuli. We documented variable complement levels in supernatants comparing secretion of left and right eye hpRPE of the same donor. In contrast to inherited eye diseases, where often both eyes are affected to a similar extent, acquired vision loss can occur in different degrees in the left and right eye. For AMD it is known that the disease progression can be dissimilar between both eyes [55,56]. Our data support this monocular specific disease progression also on the level of hpRPE cell-dependent complement secretion.

### 4.3. AMD-Risk SNPs in Complement Genes Enhanced hpRPE Cell-Dependent Complement Secretion

RPE dysfunction in AMD is associated with SNPs in complement system genes. An international AMD-genome wide association study listed six complement gene loci as top priority AMD-relevant risk candidates based on biological and statistical evidence [4,57,58]. Nevertheless, detailed studies of how precisely certain AMD-risk genotypes modify complement function and protein expression are still lacking.

Here, we found that these SNPs within the complement genes modulate local complement secretion. hpRPE obtained from donors positive for at least one homozygous complement risk SNP secreted higher levels of complement components than hpRPE cells heterozygous for the complement-associated AMD-risk SNPs. The increase was significant for alternative complement pathway components CFD/ CFI and a tendency was observed for CFH/C3. Related research showed that AMD-risk SNP enhances complement activity in the RPE and the retina. Higher levels of C5a in Bruch’s membrane and increased membrane attack complex deposition in eyes homozygous for the high-risk *CFH* genotype Y402H were identified [43,44]. Complement dysregulation was also described for a homozygous risk-SNP in the *ARMS2* gene [59]. In contrast, blood-derived CFI was reduced in AMD patients with a *CFI* SNP compared to patients without the SNP [60].

At least two mechanistic models are possible how genetic polymorphism in genes of the complement system could modify the ability of hpRPE cells to secrete components: (i) Polymorphism within a gene or in a regulatory region near a gene itself could results in gain- or loss-of transcription/secretion/function of the affected complement component. This is already known for SNPs in C3 [61,62]. (ii) Autocrine regulation of complement transcripts/secretion by complement complexes or cleavage products, which are only produced if the SNP is present, could be imaginable. Complement components or complement cleavage products can activate cellular signaling pathways via specific receptors [63,64,65], which can result in increased or reduced transcription of complement components by associated transcription factors [66,67,68]. As we showed the secretion of complement components and presents of complement activation products as well as of receptors in hpRPE cells an autocrine regulation of complement is speculatively possible.

In sum, polymorphisms within complement genes can cause different effects in AMD patients, locally in the retina and systemically.

### 4.4. hpRPE Cell-Dependent Complement Activation

Complement components can act as signal molecules by binding to their cellular receptors [8,11]. A pivotal requirement for complement activation is the cleavage of complement components, e.g., C3/C5, into their active, receptor-binding fragments. This can be carried out either by enzyme complexes formed by different complement proteins (convertases) or by specific proteases, e.g., cathepsin L or cathepsin B [11,69]. This results in high levels of anaphylatoxins C3a and C5a, which have been associated with physiological and pathological processes in the retina [27,70,71]: Eye morphogenesis [31], sub-RPE depositions [32], pro-inflammatory RPE reaction [33,34,35], PI3/Akt-pathway activation [29], and ER stress-mediated lipid accumulation [36].

Interestingly, we detected complement activation end products C3a and C5a in untreated hpRPE cells independently of the entire complement cascade. In addition, further C3 cleavage products C3d and C3dg were found cell-associated. Considering the absence of any external complement source, these findings indicate that complement activation occurred intracellularly, originating from autonomously produced complement protein. The production of C3a and C5a has previously been described by other groups [21,26], who detected the anaphylatoxins in RPE cells following nitration of the extracellular matrix or administration of amyloid β, respectively. Activated C3b was additionally described in deposits of human fetal RPE cells cultivated without serum, indicating that a local RPE cell-associated cleavage of C3 may be efficient to excite early stages of AMD [24].

Intracellular activation of C3 has been assumed to be a general phenomenon, being observed in various cell types, including epithelial cell lines and murine RPE cells [11,27]. Autocrine effects of complement have initially been described in T-cells, where these processes were important to maintain the cell homeostasis [11,72]. Similar signaling also plays a major role in the tumor microenvironment, where it promotes tumor growth [70,73]. We previously suggested an autocrine function of complement components in ARPE-19 cells exposed to oxidative stress showing a colocalization of C3 and complement receptor 3 (CR3) [22]. Since hpRPE cells do also express the corresponding complement receptors for the autonomously produced complement activation proteins, it can be assumed that hpRPE cell can be regulated by autocrine complement-associated mechanisms, which needs to be further investigated.

### 4.5. Oxidative Stress and Properdin Altered Complement and Inflammasome Associated Expression in hpRPE Cells

AMD progresses by an interplay of genetics and cellular stress, triggered for example by oxidative stimuli [74]. We showed recently that H_2_O_2_ exposure of RPE cells resulted in increased levels of RPE cell-derived complement regulator properdin [22]. Properdin is the only known positive regulator of the complement system that stabilizes the C3-convertase [75,76]. Here we answered the question, how exogenous properdin can further influence the complement homeostasis of stressed hpRPE cells.

Notably, properdin treatment of stressed hpRPE cells resulted in an upregulation of *CFD* and *CFI* transcript levels, which could modulate the immune homeostasis in the retina: CFD and CFI are both proteases of the complement pathway. CFD cleaves Factor B and activates the alternative pathway, while CFI inactivates C3b as well as C4b [8]. In accordance with transcription data, we found also increased CFD protein concentrations in supernatants of hpRPE cells 4 h after properdin treatment. However, the levels of secreted CFI were decreased after properdin treatment, which was contradictory compared to the *CFI* mRNA levels and suggested an intracellular storage of CFI mRNA or protein in properdin-treated hpRPE cells. Intracellular storage of complement components in epithelial cells was previously associated with protection against cell death mechanisms [77].

Opposing to an upregulation of *CFI* and *CFD* mRNA expression after properdin treatment, we detected a reduction of complement transcripts *C1Q* and *C3* as well as complement receptors *C3AR*, *C5AR1* and *CD11B*. Interestingly, C3 expression was shown to be upregulated in oxidatively stressed iPSC-derived RPE and ARPE-19 cells [36,78] and previous work of our group demonstrated *C5AR1* and *CD11B* upregulation in oxidatively stressed ARPE-19 cells [22]. This suggested a properdin-mediated contra regulation of complement transcription in oxidatively stressed hpRPE cells.

In line with this, we found a reconstitutional effect of properdin treatment in stressed hpRPE cells associated with inflammasome-linked transcripts. There are different opinions about the role of NLRP3 inflammasome in RPE cells homeostasis [79,80], but certainly NLRP3 has a pro-inflammatory effect. In line with that, there is an upregulation of *NLRP3* and *IL1B* mRNA in oxidative stressed RPE cells, as we and others described in the past [22,81]. The additional exogenous properdin caused a *NLRP3* and *IL1B* mRNA reduction after 4 h. This suggested a time-dependent regulative mechanism with an early anti-inflammatory effect of properdin.

### 4.6. Time-Dependent Shift in Complement Gene Expression Levels Following Properdin Treatment of Stressed hpRPE

When treating H_2_O_2_-stressed hpRPE with exogenous properdin, early complement component mRNA expression differed from late expression. Similarly, we showed earlier that oxidative stress conditions in mouse RPE lead also to a time-dependent regulation of complement component expression [82].

Of note, in our present study transcription levels of properdin-treated cells were always reversed after 24 h compared to 4 h. Our assumption of a tightly regulated time-dependent complement expression profile in the RPE and retina was further supported by others showing that complement inhibiting components peaked simultaneously with photoreceptor apoptosis after light-induced retinal damage in rats [83]. A similar effect was observed for expression of AMD-related genes and cytokines in mice after intravenous sodium iodate injection [84].

Our data indicate a time-dependent early anti-inflammatory and late pro-inflammatory response of stressed hpRPE following properdin treatment. Our findings suggest that hpRPE cells are capable to autonomously counter regulate the effects of properdin exposure. All transcript analyses in this study were performed on hpRPE cells without an AMD-associated SNP within complement genes (donors 1–4) which suggested that healthy cells can circumvent external properdin stress. However, it is not yet known, how RPE cells that are positive for a SNP within complement genes can handle properdin stress, maybe counter regulation is not possible or exuberant. This needs to be further investigated in the future.

In the meanwhile clinical studies already indicate a potent impact of anti-Properdin antibodies in AMD patients: Intravitreal injection of 10 mg anti-Properdin antibodies reduced the lesion size in GA patients by ~16% compared to sham treated patients in phase 2 studies (not yet significant, NCT02515942). It seems to be a dose dependent reaction as a combination therapy of 5 mg anti-properdin and 5 mg anti-C5 antibodies failed to reduce lesion extension compared to the sham group (NCT02515942). However, irrespective if an anti-properdin antibody shows an effect on AMD progression in the present study, anti-properdin therapies offer the great advantage that they do not completely block the complement pathway, but instead slow down this pivotal cascade by retaining the balance of the local complement system.

## 5. Conclusions

Our results suggested that SNPs within the complement system may contribute to RPE cell-dependent local complement production in the human eye. Complement activation observed at the RPE cell layer did not rely on systemic complement components but could be modulated by external stimuli. Properdin, the only known positive regulator of the complement system, ameliorated the effects of oxidative stress-induced complement expression in early phases and increased pro-inflammatory markers in late treatment phases.

In view of the limited sample size of hpRPE cells in this study, further studies with larger cohorts from different human donor eyes are warranted to confirm the role of genotype-dependent complement production of RPE cells. Further work is also needed to distinguish the exact role of intracellular or cell-associated and blood-derived complement as well as complement activation in the retina/RPE in the development of chronic inflammatory retinal diseases such as AMD.

## Figures and Tables

**Table 1:** Human primary retinal pigment epithelium (hpRPE) cell genotyping and experimental assignment in this study.

Used Methods and Results Reference						protein _(Fig. 1B)_											
			mRNA _(Fig. 1A, 3, 5)_ 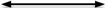				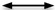										
			secretion _(Fig. 1C, 4)_ 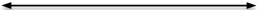									secretion _(Fig. 1C, 2, S2)_ 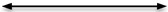					
	Gene Accession		RPE donors														
Gene	Number	SNP ID	1	2	3	4	5	6	7	8	9	10	11	12	13	14	15
*CFH*	NG_007259	rs121913059	C	C	C	C	C	C	C	C	C	C	C	C	C	C	C
*CFH_(Y402H)_*	NG_007259	rs1061170	CT	T	CT	T	C	T	CT	T	T	T	CT	T	CT	CT	CT
*CFH*	NG_007259	rs570618	GT	G	GT	G	T	G	GT	G	G	G	GT	G	GT	GT	GT
*CFH*	NG_007259	rs10922109	AC	A	AC	AC	C	AC	AC	AC	AC	A	AC	C	AC	AC	AC
*CFHR3/CFHR1*	NG_015993	rs61818925	G	GT	G	G	GT	G	GT	G	G	G	G	G	G	G	G
*C3*	NG_009557	rs2230199	C	C	C	C	C	C	C	C	C	C	C	C	CG	C	C
*C3*	NG_009557	rs147859257	T	T	T	T	T	T	T	T	T	T	T	T	T	T	T
*C2/CFB*	NG_011730	rs116503776	G	GA	G	G	G	G	GA	G	G	G	G	GA	A	G	G
*C2/CFB*	NG_011730	rs144629244	C	C	C	C	C	C	C	C	C	C	C	C	C	C	C
*CFI*	NG_007569	rs141853578	C	C	C	C	C	C	C	C	C	C	C	C	C	C	C
*CFI*	NG_007569	rs10033900	TC	T	TC	TC	T	TC	TC	TC	T	TC	TC	TC	C	TC	C
*ARMS2*	NG_011725	rs3750846	A	AG	AG	A	AG	A	A	AG	AG	A	AG	AG	AG	A	A
*C9*	NG_009894	rs6235861	G	G	G	G	G	G	G	G	G	G	G	G	G	G	G
	none AMD-risk						heterozygous								AMD-risk		

## Supplementary Materials

The following are available online at https://www.mdpi.com/2076-3921/9/9/793/s1, Figure S1: Ex vivo cultivated human hpRPE cells were pigmented and vital for 14 days on transwell filters, Figure S2: Western Blots of C3a and C5a in hpRPE cells, Figure S3: Complement component secretion is different in hpRPE cells from left and right donor eyes, Table S1: In-house designed RT-qPCR and PCR primers, Table S2: QuantiTect PrimerAssays (Qiagen).
