# Supplementary material for: Properdin Modulates Complement Component Production in Stressed Human Primary Retinal Pigment Epithelium Cells
Source: Antioxidants (Basel). 2020 Aug 26;9(9):793. doi: 10.3390/antiox9090793 (PMC7555107; doi:10.3390/antiox9090793)
Supplement: Supplementary file 1 [file antioxidants-09-00793-s001.pdf]

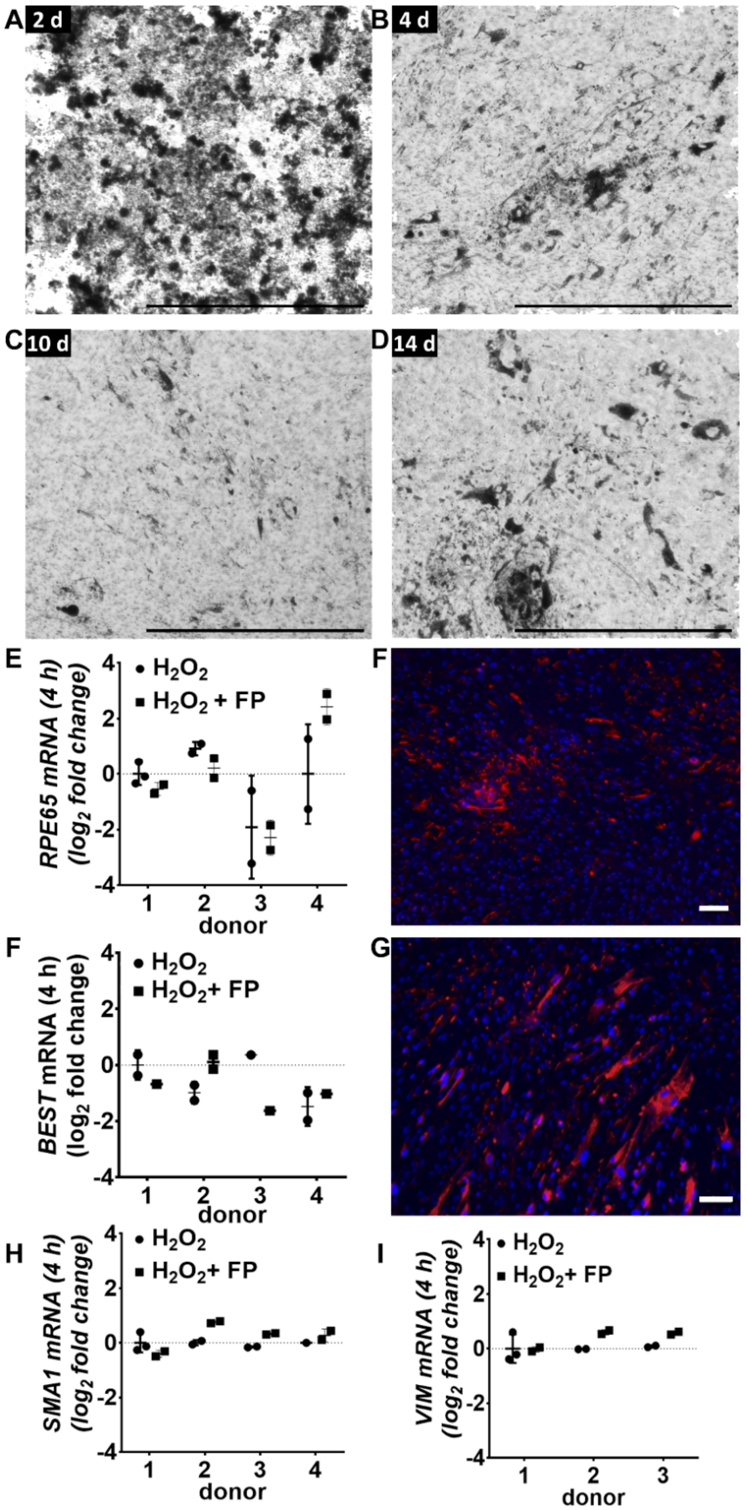

Figure S1. *Ex vivo* cultivated human hpRPE cells show RPE-specific markers. (**A-D**) hpRPE were pigmented and vital for 14 days on transwell filters. Shown are the cells of donor 2 at day (**A**) 2, (**B**) 4, (**C**) 10 and (**D**) 14 as an example. Scale bars 400 µm. Magnification 10x. (**E-F**) hpRPE were positive for (**E**) *RPE65* mRNA and (**F**) RPE65 protein in immunostainings. (**G-H**) hpRPE were positive for (**G**) Bestrophin (*BEST*) mRNA and (**H**) bestrophin protein in immunostainings. (**F, H**) Shown are representative examples after 10 days in culture. Scale bars 100 µm. Magnification 100x. (**I, J**) Marker for epithelial–mesenchymal transition (**I**) mRNA *SMA1* and (**J**) mRNA *VIM* were partly increased after properdin treatment of stressed hpRPE cells.

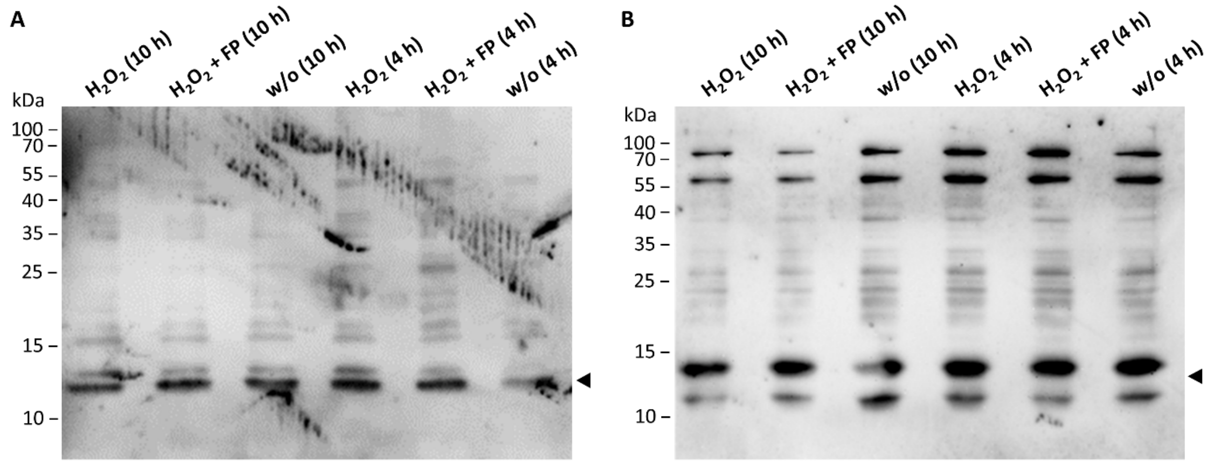

Figure S2. Western Blots of C3a and C5a in hpRPE cells. (**A**) C3a was detected in 5 h and 10 h stressed (H_2O_2, H_2O_2 + FP) and unstressed (w/o) hpRPE cells at approx. 12 kDa (arrow). Exemplarily shown for donor 6. (**B**) C5a was detected as double band in 5 h and 10 h stressed (H_2O_2, H_2O_2 + FP) and unstressed (w/o) hpRPE cells between 10 – 15 kDa (arrow). Exemplarily shown for donor 5. FP - properdin. Reduced conditions.

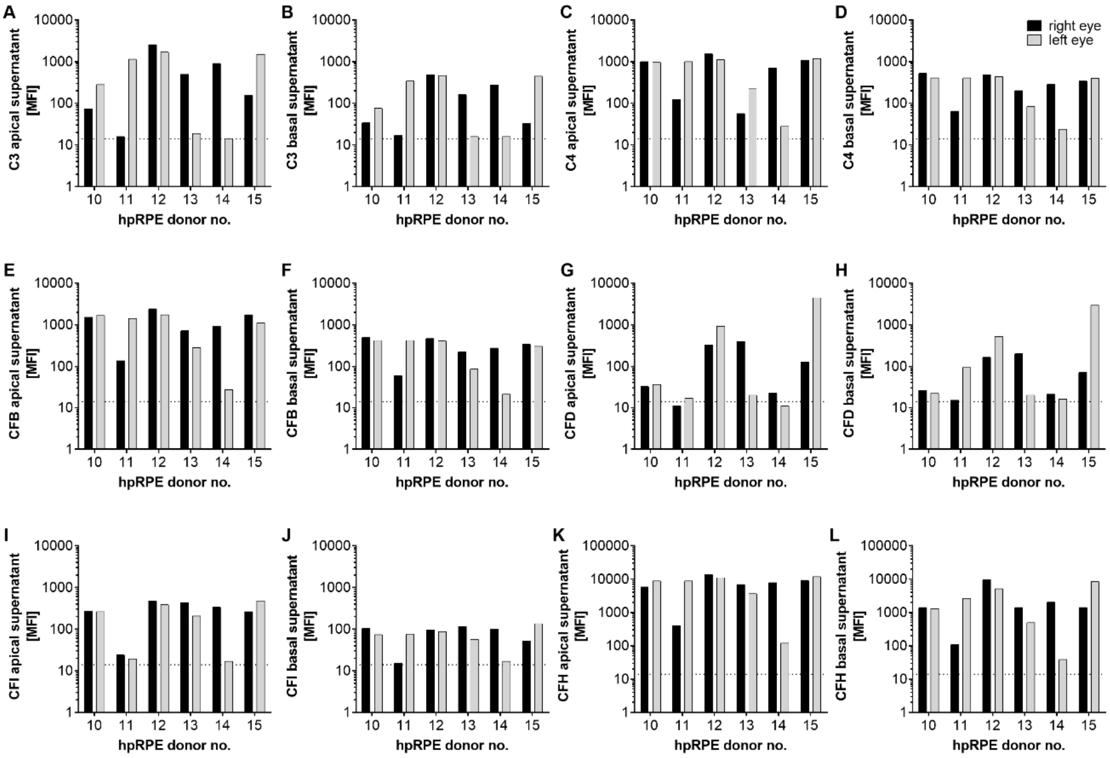

Figure S3. Complement component secretion is different in hpRPE cells from left and right donor eyes. Complement secretion of (**A, B**) C3, (**C, D**) C4, (**E, F**) CFB, (**G, H**) CFD, (**I, J**) CFI and (**K, L**) CFH was increased at the (**A, C, E, G, I, K**) apical compared to the (**B, D, F, H, J, L**) basal side. hpRPE isolated from the right eye (black) showed different complement component secretion activity than hpRPE from the left eye (grey). Dotted line shows blank control.

Table S1. In-house designed PCR primers.

transcript	gene accession number	name of sequence	application
ARMS_375	NG_011725	fw: CATTTCTAATATCAAATCAAAACATTCAT<br>rv: CCTGGGGCTCTGTTTGAATTG	
C2_116	NG_011730	fw: AGCACCATCTACACTTCGCC<br>rv: CATGGCCCTTACCTCGACAT	
C2_144	NG_011730	fw: CAGGGGGAGATCAGAATCGTC<br>rv: GTGGGGGCAGGGAAATTCTTT	
C3_147	NG_009557	fw: TTGCCTCTCCTAAGCCTGTG<br>rv: GGCTAGGGTCTCACGAGG	
C3_223_2	NG_009557	fw: CTGGATGAAGAGGTACCCGC<br>rv: CTTGGAACAGACCCCTGACA	
C9_623	NG_009894	fw: TCTGTGACAATGGCAGACAACT<br>rv: TTTATTCCCCACTCTCTGTTATCTT	
CFH_106	NG_007259	fw: CCTTTGTTAGTAACTTTAGTTCGTCTT<br>rv: GGAGTAGGAGACCAGCCATTC	hpRPE genotyping (2.2; Tab. 1)
CFH_109	NG_007259	fw: AGTCTACCTTGCTAACGGTTC<br>rv: CCTTCTAGATTGTAGTACCTGTTGCT	
CFH_121	NG_007259	fw: CAGATCCGTGTGTAATATCCCGA<br>rv: TAAGAAGAGAGCCACCGGTCTCA	
CFH_570	NG_007259	fw: AGTGGTGAGGAAAAATGTAGAATGT<br>rv: CATCTCCATCTTTCTTAAATTCTGTGA	
*CFH_618**	NG_007259	fw: GTGCATTCAGGGGCATATTC<br>rv: CCTGAAGACACAAATCTCACTGG	
CFI_100	NG_007569	fw: CCTGCAGTGTGGTCTGTGAG<br>rv: CCAGTGCTACAAGGTGGGAA	
CFI_141	NG_007569	fw: ATCCACTGATACAAGCGCTCA<br>rv: TTAAAATCGTCATGATGTTCAAAGC	
IL1B	NG_008851	fw: CTCGCCAGTGAAATGATGGCT<br>rv: GTCGGAGATTCGTAGCTGGAT	PCR (2.2/3; Fig. 5A) and RT-qPCR (2.3; Fig. 5B, C)
SMA1	NG_006672	fw: GCCTTGGTGTGTGACAATGG<br>rv: AAAACAGCCCTGGGAGCAT	qPCR (2.3; Fig. S1)
VIM	NG_012413	fw: TGTCCAAATCGATGTGGATGTTTC<br>rv: TTGTACCATTCTTCTGCCTCCTG	

*transcript refers to SNP *CFHR3/1_618*

Table S2 QuantiTect PrimerAssays (Qiagen)

mRNA transcript	gene accession number	PrimerAssay name	catalogue number
GAPDH	NG_007073	Hs_GAPDH_1_SG	QT00079247
C1Q	NG_007283	Hs_C1QB_1_SG	QT00003493
C3	NG_009557	Hs_C3_1_SG	QT00089698
C4A	NG_011638	Hs_C4A_1_SG	QT00237160
C4B	NG_011639	Hs_C4B_1_SG	QT00237167
C5	NG_007364	Hs_C5_1_SG	QT00088011
CFB	NG_008191	Hs_BF_1_SG	QT00012138
CFD	NG_007274	Hs_CFD_1_SG	QT00212191
CFI	NG_007569	Hs_CFI_1_SG	QT00213794
CFH	NG_007259	Hs_CFH_1_SG	QT00001624
CFP	NG_009893	Hs_CFP_1_SG	QT00010514
CD46	NG_009296	Hs_MCP_1_SG	QT00073689
CD59	NG_008057	Hs_CD59_1_SG	QT00035952
C3AR	NG_050736	Hs_C3AR1_1_SG	QT00090398
C5AR1	NM_001736.4	Hs_C5R1_1_SG	QT00997766
CD11B	NG_011719	Hs_ITGAM_1_SG	QT00031500
NLRP3	NG_007509	Hs_NLRP3_1_SG	QT00029771
RPE65	NG_008472	Hs_RPE65_1_SG	QT00001351
BEST	NG_009033	Hs_BEST1_1_SG	QT00023282
